# Transcranial direct current stimulation for migraine: a systematic review and meta‐analysis of randomized controlled trials

**DOI:** 10.1111/cns.13843

**Published:** 2022-04-19

**Authors:** Peiwei Hong, Yao Liu, Yang Wan, Hai Xiong, Yanming Xu

**Affiliations:** ^1^ Department of Geriatric Medicine and Neurology West China School of Public Health and West China Fourth Hospital Sichuan University Chengdu People’s Republic of China; ^2^ Department of Neurology West China Hospital Sichuan University Chengdu People’s Republic of China; ^3^ Xindu Hospital of Traditional Chinese Medicine Chengdu Medical College Chengdu Sichuan People’s Republic of China; ^4^ Medical College of Tibet University Lhasa People’s Republic of China

**Keywords:** efficacy, meta‐analysis, migraine, safety, tDCS, transcranial direct current stimulation

## Abstract

**Background:**

Transcranial direct current stimulation (tDCS) is a promising method for migraine treatment. In this study, we investigated the efficacy and safety of tDCS for migraine by conducting a systematic review and meta‐analysis of randomized controlled trials (RCTs).

**Methods:**

We searched PubMed, EMBASE, Cochrane Library, and Web of Science up to December 02, 2021 for RCTs reporting tDCS for migraine treatment. Two authors independently evaluated the eligibility of the retrieved trials and extracted relevant data. Outcomes for the quantitative synthesis were reduction in migraine days per month and adverse events.

**Results:**

Eleven RCTs that included 425 patients with migraine were evaluated in the meta‐analysis. The efficacy and safety of anodal or cathodal stimulation targeting different brain areas, including primary motor cortex (M1), primary sensory cortex (S1), dorsolateral prefrontal cortex (DLPFC), and visual cortex (VC), were assessed in the RCTs enrolled. We found that tDCS with M1 and VC activation could reduce No. of migraine days per month in patients with migraine. Meanwhile, tDCS with VC inhibition could also reduce No. of migraine days per month in patients with migraine. However, there were no differences in the incidence of adverse events between the two groups.

**Conclusion:**

tDCS activates M1 or activates/inhibits VC which could improve migraine symptoms. tDCS is an effective, preventive, and safe treatment for migraine.

## Graphical abstract

The figure presents reduction of No. of migraine days per month from baseline to different post‐treatment period: (A) no more than 1 month, (B) more than 1 month and no more than 3 months, (C) more than 3 months. And we found that tDCS with activating M1 and VC could reduce No. of migraine days per month in patients with migraine. And tDCS with inhibiting VC could reduce No. of migraine days per month in patients with migraine.

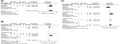

## 1 BACKGROUND

Migraine is the most prevalent neurological disorder worldwide, and more than 1 billion people have migraine according to the Global Burden of Disease study in 2016.^1^ The typical characteristic feature is recurrent headaches lasting for 4–72 h, occurring at a unilateral location, pulsating quality, and moderate or severe intensity. It is associated with nausea, phonophobia, and photophobia according to the International Classification of Headache Disorders, 3rd edition (ICHD‐3).^2^ Although numerous pharmacological treatments are available, including triptans, and drug target calcitonin gene‐related peptides (CGRP), their effectiveness, and safety are only partial.^3^ Furthermore, some migraineurs are hesitant to choose pharmacological treatment.

Noninvasive brain stimulation (NIBS), which targets either transcutaneous peripheral nerves or the brain, is a much better tolerated treatment for migraine, some of which are recommended in guidelines.^4^, ^5^ Transcranial direct current stimulation (tDCS) is an effective method to inhibit or activate the underlying cerebral cortex, thereby regulating the abnormal cortico‐thalamic information processing in migraine.^6^ However, the target brain area, sessions, and results of clinical trials are varied.^7^, ^8^, ^9^, ^10^, ^11^, ^12^, ^13^, ^14^, ^15^, ^16^, ^17^, ^18^, ^19^ Hence, we conducted a systematic review and meta‐analysis to evaluate the efficacy and safety of tDCS for migraine.

## 2 METHODS

### 2.1 Literature search

Relevant literature was identified from four electronic databases: PubMed, EMBASE, Cochrane Library, and Web of Science. The search dates were inception to December 02, 2021. The terms used for searching tDCS literature were “transcranial direct current stimulation” and “tDCS”. The terms used for searching migraine literature were “migraine disorders,” “migraine without aura,” and “migraine with aura.” The search strategies are summarized in Table S1.

### 2.2 Inclusion and exclusion criteria

The PICOS framework was used to organize the inclusion criteria. Population (P): studies that enrolled participants with migraine; intervention (I): tDCS; comparison (C): sham stimulation; outcomes (O): number of migraine days per month or pain intensity; study design (S): randomized controlled trials.

Studies that met any of the following criteria were excluded: (1) participants included patients with other headache disorders or healthy volunteers; (2) studies were published as conference abstracts without sufficient data to calculate the effect size.

### 2.3 Data extraction and analysis

Two authors screened the literature, read full‐text articles, and decided to enroll independently. If there was disagreement, then the decision was made by a third author. Data extraction and handling of missing values have been described in our previous publication.^20^ The quality and risk of bias of studies with randomized controlled trial designs were assessed using the Cochrane Handbook's tool for assessing the risk of bias. The primary outcomes were the reduction in number of migraine days per month from baseline to post‐treatment. Secondary outcomes were the reduction in pain intensity and the incidence of adverse events.

### 2.4 Statistical analysis

For statistical analysis, Review Manager 5.3 (Cochrane Collaboration, http://tech.cochrane.org/home) was used. The heterogeneity of the enrolled trials was evaluated using *I* ^2^. If *I* ^2^ was <50%, then the heterogeneity of the trials enrolled was deemed acceptable and the differences between groups were analyzed using the fixed‐effects model. Otherwise, a random‐effects model was used to eliminate the effect of heterogeneity and draw conclusions. The inverse variance method was used to measure the difference between continuous variables in the enrolled trials and weighted mean difference (WMD). Odds ratios (OR) were calculated for dichotomous variables, and the Mantel–Haenszel test was used to assess the difference. The significant level was set at *p *< 0.05.

## 3 RESULTS

### 3.1 Literatures screening and risk of bias assessment

**FIGURE 1:**
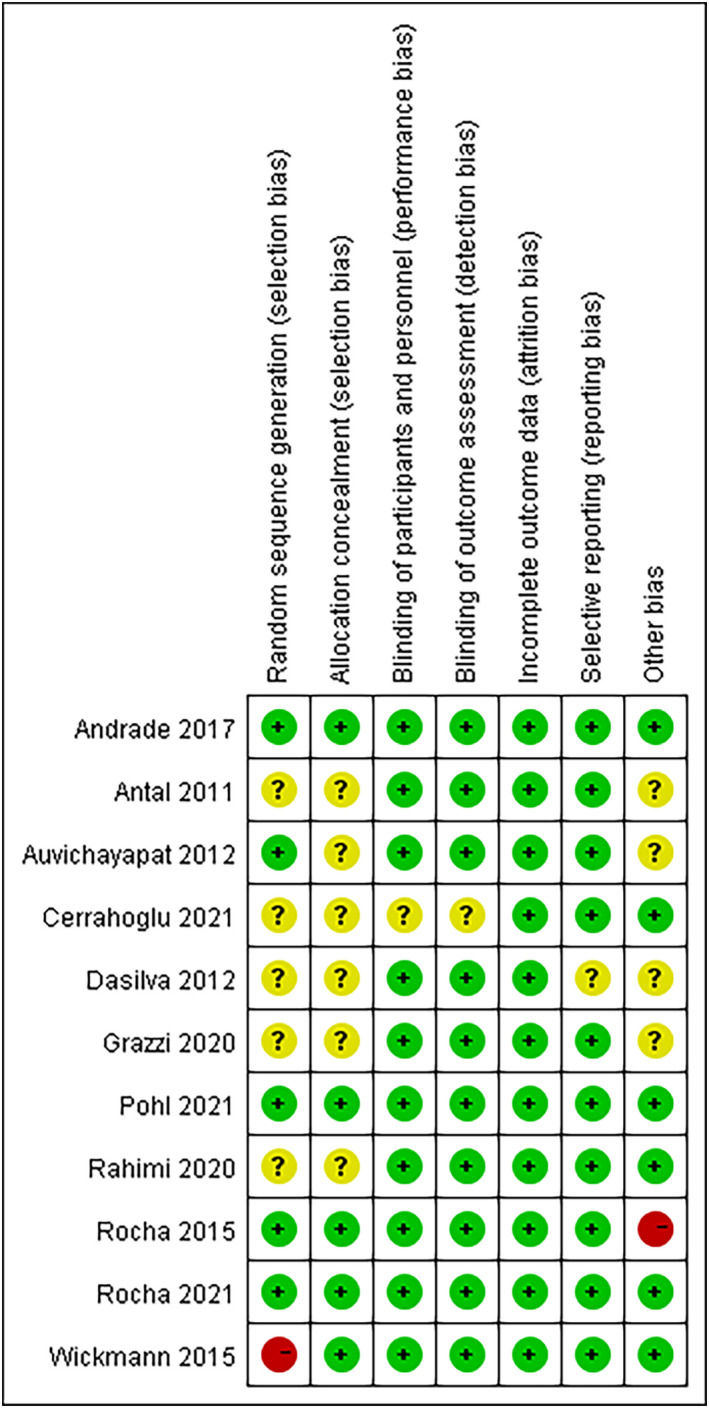
Risk of bias in the trials enrolled. The risk of bias of in the enrolled trials was judged by the authors. The circles in red, yellow, and green indicate high, unclear, and low risk, respectively

Forty‐five records were identified according to our search strategy. Twelve studies with 11 independent trials were included in accordance with our inclusion criteria. A flowchart of literature screening is shown in Figure S1. The quality of the trials enrolled was evaluated by risk of bias, and we found that a high risk existed in one term of two trials, low risk existed in all terms of three trials, and some terms of trials had unclear risk because of insufficient information (Figure 1).

### 3.2 Characteristics of the trials enrolled

The 11 enrolled trials included participants with episodic migraine with or without aura, chronic migraine, and menstrual migraine, which were diagnosed according to ICHD‐2 or ICHD‐3. Anodal and cathodal stimulations were applied to 7 and 6 trials, respectively. The stimulation targeted different brain areas, including primary motor cortex (M1), primary sensory cortex (S1), dorsolateral prefrontal cortex (DLPFC), and visual cortex (VC). Seven different treatment methods, namely anodal DLPFC, anodal M1, cathodal M1, cathodal S1, anodal VC, cathodal VC, and sham, were applied to the 11 trials enrolled. A total of 425 patients with migraine were enrolled in the 11 trials. The numbers of patients in the anodal DLPFC, anodal M1, cathodal M1, cathodal S1, anodal VC, cathodal VC and sham was 3, 115, 59, 15, 11, 44, and 178, respectively. The intensity and total dosage of stimulation were varied, as summarized in Table 1. The assessment time points ranged from immediate post‐treatment assessment to 12 months post‐treatment assessment.

**TABLE 1:** Characteristic of trials enrolled

Study ID	Participant	Group allocation	Brain target	Intensity	Total dose
Antal 2011^16^	Migraine with/without aura; chronic migraine	Cathodal VC = 15 Sham = 15	VC	1 mA for 15 min once daily	3 days/week for 3 weeks
Auvichayapat 2012^8^	Episodic migraine	Anodal M1 = 20 Sham = 17	LM1	0.029 and 0.08 mA/cm^2^ for 20 min once daily	20 consecutive days
Dasilva 2012^9^	Chronic migraine	Anodal M1 = 8 Sham = 5	M1	2 mA for 20 min once daily	Every other day during weekdays for 4 weeks
Rocha 2015^17^	Migraine with/without aura	Cathodal VC = 10 Sham = 5	VC	2 mA for 20 min once daily	3 days/week for 4 weeks
Wickmann 2015^18^	Menstrual migraine	Cathodal VC = 8 Sham = 8	VC	2 mA for 20 min once daily	5 days before the expected onset of the menstruation for 12 weeks
Andrade 2017^7^	Chronic migraine	Anodal M1 = 6 Anodal DLPFC = 3 Sham = 4	LM1 DLPFC	2 mA for 20 min once daily	Three times per week for 1 month
Grazzi 2020^11^	Chronic migraine	Anodal M1 = 45 Cathoda M1 = 44 Sham = 46	RM1	2 mA for 20 min once daily	Five consecutive days
Rahimi 2020^15^	Migraine	Cathodal M1 = 15 Cathodal S1 = 15 Sham = 15	RM1 (C4) RS1	1 mA for 20 min once daily	3 days/week for 5 weeks, 2 days/weeks for 2 weeks, 1 day/week for 3 weeks
Cerrahoglu 2021^12^	Migraine	Anodal M1 = 36 Sham = 41	LM1 (C3)	2 mA for 20 min once daily	Three consecutive days
Pohl 2021^14^	Episodic migraine	Anodal VC = 11 Sham = 12	VC	1 mA for 20 min once daily	4 weeks
Rocha 2021^19^	Migraine	Cathodal VC = 11 Sham = 10	VC	2 mA for 20 min once daily	3 days/week for 4 weeks

Abbreviations: M1, primary motor cortex; DLPFC, dorsolateral prefrontal cortex; S1, primary sensory cortex; VC, visual cortex; RM1, right primary motor cortex; LM1, left primary motor cortex; RS1, right primary sensory cortex.

### 3.3 Reduction in the number of migraine days per month from baseline to post‐treatment

**FIGURE 2:**
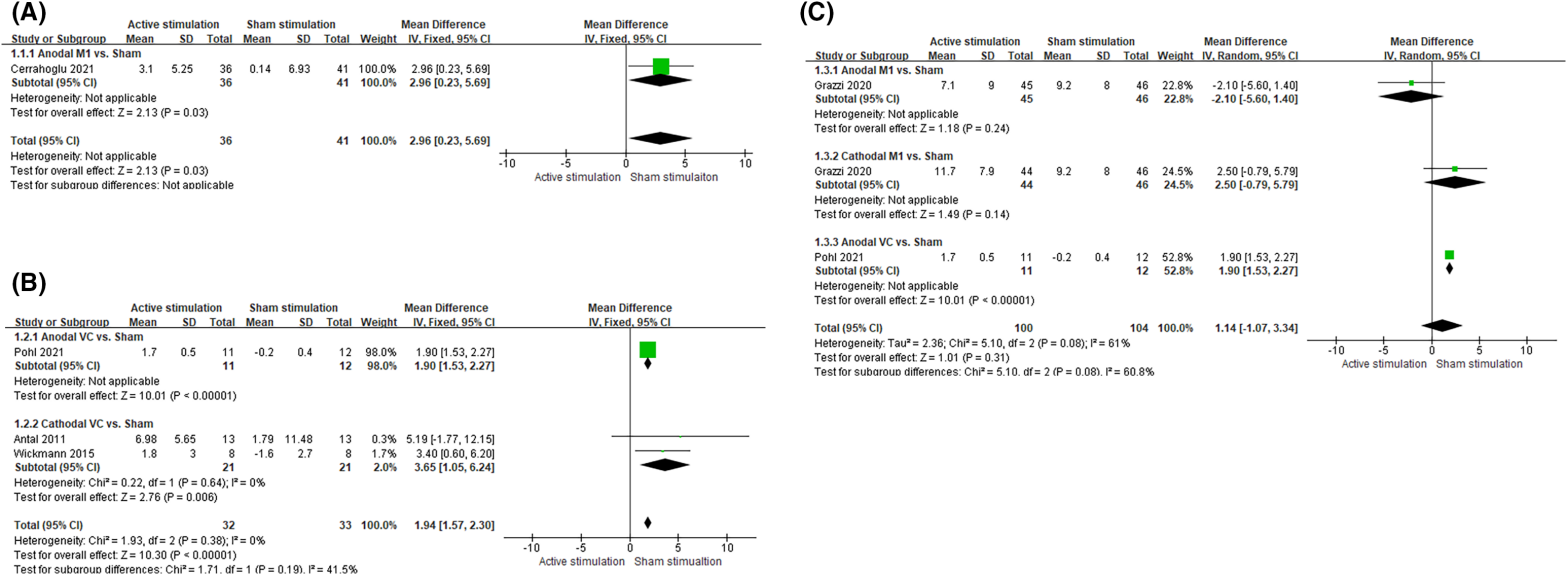
Reduction in the number of migraine days per month from baseline to post‐treatment. The figure shows a reduction in the number of migraine days per month from baseline to different post‐treatment periods: (A) no more than 1 month, (B) more than 1 month and no more than 3 months, and (C) more than 3 months

Five trials explored the effect of active stimulation to reduce the number of migraine days per month. We found that active stimulation achieved a significant reduction in the post‐treatment period of no more than 1 month (WMD = 2.96, 95% confidence interval [CI] = [0.23, 5.69], *I* ^2^ = not applicable, *p* = 0.03), and more than 1 month and no more than 3 months (WMD = 1.94, 95% CI = [1.57, 2.30], *I* ^2^ = 0%, *p *< 0.00001), as compared with sham stimulation. However, in a period of more than 3 months, there was no difference between the two groups (WMD = 1.14, 95% CI = [−1.07, 3.34], *I* ^2^ = 61%, *p* = 0.31) (Figure 2).

Subgroup analysis revealed that anodal M1 achieved a significant reduction in the post‐treatment period of no more than 1 month (Figure 2A). Meanwhile, anodal and cathodal VC achieved a significant reduction in the post‐treatment period of more than 1 month and no more than 3 months (Figure 2B). Anodal VC was significantly reduced in the post‐treatment period of more than 3 months (Figure 2C). However, anodal M1 and cathodal M1 did not reduce the number of migraine days per month in the post‐treatment period of more than 3 months (Figure 2C).

### 3.4 Reduction in pain intensity from baseline to post‐treatment

**FIGURE 3:**
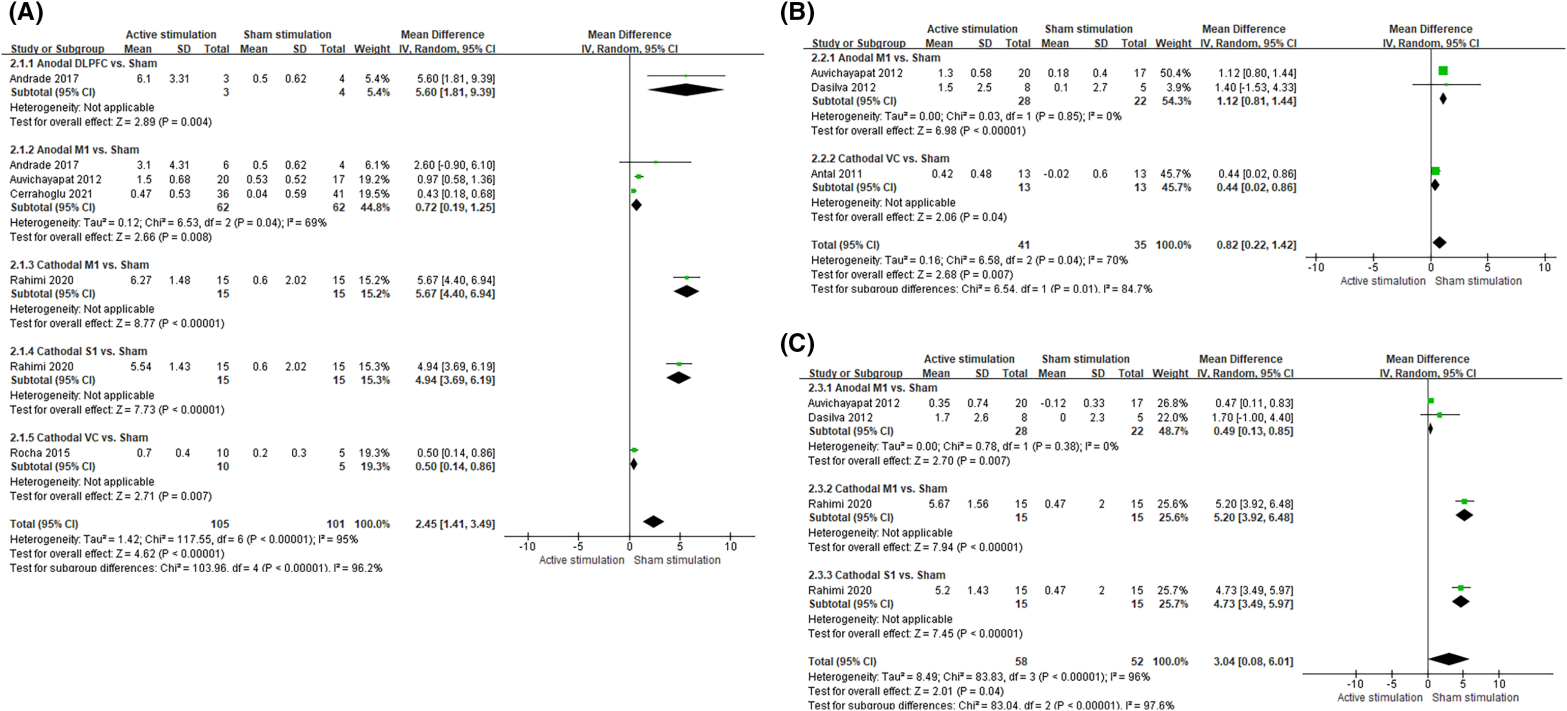
Reduction in pain intensity from baseline to post‐treatment. The figure presents the reduction in pain intensity from baseline to different post‐treatment periods: (A) no more than 1 month, (B) more than 1 month and no more than 3 months, and (C) more than 3 months

Seven trials with high heterogenicity explored the effect of active stimulation to reduce the pain intensity. We found that active stimulation achieved a significant reduction in the post‐treatment period of no more than 1 month (WMD = 2.45, 95% CI = [1.41, 3.49], *I* ^2^ = 95%, *p *< 0.00001), more than 1 month and no more than 3 months (WMD = 0.82, 95% CI = [0.22, 1.42], *I* ^2^ = 70%, *p* =0.007), and more than 3 months (WMD = 3.04, 95% CI = [0.08, 6.01], *I* ^2^ = 95%, *p* = 0.04), as compared with sham stimulation (Figure 3).

Subgroup analysis found that anodal M1, cathodal M1, cathodal S1, and cathodal VC achieved a significant reduction in the post‐treatment period of no more than 1 month (Figure 3A). Meanwhile, anodal M1 and cathodal VC achieved a significant reduction in the post‐treatment period of more than 1 month and no more than 3 months (Figure 3B). Anodal M1, cathodal M1, and cathodal S1 achieved a significant reduction in the post‐treatment period of more than 3 months (Figure 3C).

### 3.5 Safety of active stimulation

In the enrolled trials, patients treated with active stimulation experienced burning sensations, dizziness, drowsiness, fatigue, headache, itching, nausea, pain, skin redness, and tingling. However, there were no differences in the incidence of these adverse events between active and sham stimulation according to the meta‐analysis (Table 2).

**TABLE 2:** Adverse events

	Number of trials	Active stimulation		Sham stimulation		Effect estimate		
		*n*	*N*	*n*	*N*	OR 95% CI	*I* ^2^	*p*
Burning sensation	4	15	75	3	67	3.16 [0.93, 10.78]	21%	0.07
Dizziness	2	4	29	3	21	0.53 [0.08, 3.40]	0%	0.5
Drowsiness	4	11	47	8	31	0.70 [0.24, 2.02]	54%	0.69
Fatigue	2	8	24	8	25	1.08 [0.33, 3.60]	46%	0.89
Headache	6	30	90	16	59	1.39 [0.58, 3.33]	0%	0.46
Itching	5	20	87	14	84	1.33 [0.56, 3.17]	8%	0.52
Nausea	2	5	47	1	53	4.85 [0.72, 32.59]	0%	0.1
Pain	3	14	29	10	22	1.06 [0.33, 3.37]	0%	0.93
Skin redness	2	8	18	4	10	1.14 [0.29, 4.41]	84%	0.98
Tingling	8	53	125	43	103	1.01 [0.54, 1.86]	0%	0.98

Abbreviations: OR, odds ratio; CI, confidence interval.

## 4 DISCUSSION

Our review demonstrates that active stimulation with tDCS can reduce the number of migraine days per month or pain intensity in patients with migraine. Moreover, tDCS with M1 and VC activation reduced the number of migraine days per month in patients with migraine. tDCS with activation of DLPFC and M1 could improve migraine pain intensity. Meanwhile, tDCS with VC inhibition could reduce the number of migraine days per month in patients with migraine. tDCS with inhibition of M1, S1 and VC could reduce pain intensity in migraine patients. However, tDCS with inhibition of M1 did not reduce the number of migraine days per month in the post‐treatment period of more than 3 months. Active stimulation with tDCS did not increase the incidence of adverse events. Therefore, tDCS is an effective and safety option for preventive treatment of migraine.

Abnormal cortico‐thalamic information processing, characterized by a normal‐to‐low amplitude response to low numbers of stimuli, followed by an amplitude increase during prolonged stimulation between attacks, is a characteristic of migraineurs’ brain.^6^ Moreover, changes in glutamatergic function and homeostatic plasticity appear to be associated with cortical excitability disorders.^7^ M1 and S1 have been considered the central locus for pain control in some conditions; however, the mechanisms associated with M1 and S1 activation appear to involve other cortical areas, such as DLPFC, thalamus, cerebellum, and anterior cingulate cortex.^7^, ^15^ Anodal and cathodal tDCS can modify the cortical spreading depression, which is important for migraine pathophysiology and abnormal ion homeostasis.^7^, ^15^ Previous studies found that the frequency of headache and pain intensity was reduced by excitatory NIBS in the M1 or DLPFC. However, inhibitory NIBS on the vertex or VC did not significantly change the pain intensity or frequency of headache attacks in migraineurs.^21^, ^22^ Our results showed that either activating or inhibiting M1 or VC could improve migraine prognosis. Meanwhile, activating the DLPFC or inhibiting S1 could improve migraine prognosis.

A previous study performed a meta‐analysis of the therapeutic effects of different NIBS in stimulating a particular brain region in patients with migraine.^21^ Another study systemically reviewed the therapeutic effects of tDCS in different brain regions with seven clinical trials.^22^ This study focused on the tDCS targeting different brain areas, and 11 clinical trials were enrolled. We found that tDCS stimulated M1, S1, DLPFC, and VC to relieve pain intensity.

Neurovascular mechanisms of migraine include that activation of the trigeminovascular system, which can cause the release of vasoactive neuropeptides, subsequently leading to plasma protein extravasation, acute neurogenic inflammation, and transient vasodilation of the vessels.^23^, ^24^, ^25^ Alteration of cerebrovascular function and the decrease in endothelial shear stress on magnetic resonance imaging (MRI) have proven this hypothesis.^26^, ^27^ Furthermore, the neurovascular mechanism is a potentially important therapeutic target for the treatment of migraine, which has been proven by the successful application of triptans and CGRP antagonists.^25^ Previous studies have found disparities in cerebrovascular diseases, which are mainly caused by sex difference.^28^, ^29^ Transcranial alternating current stimulation can modulate pain empathy in a sex‐dependent manner.^30^ In our study, we did not observe any effect of tDCS, stratified by sex. Therefore, it is important that future studies should consider the impact of sex differences in tDCS for migraine.

The limitations of this study are as follows: First, the sample size of the majority of the trials enrolled was small, and estimation of the effect size may have been underpowered. Second, although the enrolled patients were migraineurs, the subtypes of migraine were not distinguished, which might tamper with the validity of our findings. Third, fewer studies were included in each subgroup, which resulted in the reproducibility of each experiment not being verified. Finally, our results could not be applied to the abortive treatment of migraine because of the absence of outcomes such as pain‐free 2 h or pain‐relief 2 h. Hence, further studies with a larger sample size, uniform migraine, multicenter, and different treatment goals must be conducted to evaluate the efficacy and safety of tDCS targeting different brain areas.

## 5 CONCLUSIONS

tDCS activates M1, or activates/inhibits VC. which could improve migraine symptoms. tDCS is an effective preventive and safe treatment for migraine.

## CONFLICT OF INTEREST

The authors declare that they have no competing interests.

## AUTHOR CONTRIBUTIONS

Xu Y, Xiong H and Wan Y put forward the idea; Hong P and Liu Y acquired the data. Hong P and Liu Y analyzed the data. Xu Y wrote the first draft; Xiong H, Wang Y, Hong P, and Liu Y revised the draft. All authors have approved the final manuscript.

## Supporting information

Fig S1

Table S1

## Data Availability

All data generated or analyzed during this study are included in this published article and its supplementary information files.
