# Supplementary material for: Transcranial direct current stimulation for migraine: a systematic review and meta‐analysis of randomized controlled trials
Source: CNS Neurosci Ther. 2022 Apr 19;28(7):992–8. doi: 10.1111/cns.13843 (PMC9160451; doi:10.1111/cns.13843)
Supplement: Supplementary file 1 — Fig S1 [file CNS-28-992-s001.docx]

**Identification of studies via other methods**

**Identification of studies via databases and registers**

Records identified from:

Websites (n =0 )

Organisations (n = 0)

Citation searching (n = 1)

etc.

Records removed *before screening*:

Duplicate records removed (n = 17)

Records marked as ineligible by automation tools (n = 0)

Records removed for other reasons (n = 0)

Records identified from*:

Databases (n = 44)

Registers (n = 0)

**Identification**

Records screened

(n = 27)

Records excluded**

(n =11 )

Reports not retrieved

(n =0 )

Reports sought for retrieval

(n =1 )

Reports sought for retrieval

(n =16 )

Reports not retrieved

(n = 2)

**Screening**

Reports assessed for eligibility

(n =0)

Reports excluded:

Comparison wasn’t sham stimulation (n =1)

Reports assessed for eligibility

(n =14 )

Reports excluded:

Other headache disorder (n = 1)

Comparison wasn’t sham stimulation (n=1)

Studies included in review

(n =11 )

Reports of included studies

(n = 12)

**Included**

*Consider, if feasible to do so, reporting the number of records identified from each database or register searched (rather than the total number across all databases/registers).

**If automation tools were used, indicate how many records were excluded by a human and how many were excluded by automation tools.

*From:*  Page MJ, McKenzie JE, Bossuyt PM, Boutron I, Hoffmann TC, Mulrow CD, et al. The PRISMA 2020 statement: an updated guideline for reporting systematic reviews. BMJ 2021;372:n71. doi: 10.1136/bmj.n71. For more information, visit: <http://www.prisma-statement.org/>
