# Supplementary material for: Transcranial direct current stimulation for migraine: a systematic review and meta‐analysis of randomized controlled trials
Source: CNS Neurosci Ther. 2022 Apr 19;28(7):992–8. doi: 10.1111/cns.13843 (PMC9160451; doi:10.1111/cns.13843)
Supplement: Supplementary file 2 — Table S1 [file CNS-28-992-s002.docx]

**Table S1 Search strategy**

| Database | Search strategy | Result |
| --- | --- | --- |
| PUBMED | (tDCS) OR ("Transcranial Direct Current Stimulation"[Mesh])) AND ("Migraine Disorders"[Mesh] OR "Migraine without Aura"[Mesh] OR "Migraine with Aura"[Mesh]) Filter: clinical trial | N=15 |
| Embase | #1 familial hemiplegic migraine/ or transformed migraine/ or migraine/ or hemiplegic migraine/ or episodic migraine/ or "MIDAS (migraine)"/ or retinal migraine/ or migraine aura/ or basilar type migraine/ or menstrual migraine/ or sporadic hemiplegic migraine/ or vestibular migraine/ or migraine with aura/ or complicated migraine/ or migraine without aura/ （N=71410）  #2 transcranial direct current stimulation/ (N=9007)  #3 clinical trial/(N=1029323)  #4 #1 and #2 and #3 (N=7) | N=7（原来6） |
| Cochrane Library | #1 migraine disorders (N=2706)  #2 transcranial direct current stimulation.mp.(N=4528)  #3 clinical trial.mp. or exp Clinical Trial/ (n= 454824)  #4 #1 and #2 and #3 (n=3) | N=3 |
| Web of science | #1 Migraine Disorders (topic) or Migraine without Aura (topic) or Migraine with Aura (topic) or migraine (topic) (N=88499)  #2 transcranial direct current stimulation (topic) or tDCS (topic) (n=11047)  #3 #1 and #2 Filter: clinical trail (n=19) | N=19 |
